# Dissecting the Radioresponse Heterogeneity of Lung Adenocarcinoma Cells From the Perspective of Transcriptional and Regulatory Landscape

**DOI:** 10.1002/mco2.71071

**Published:** 2026-10-21

**Authors:** Huan Tao, Yaru Li, Leixin Ge, Lu Chen, Yang Zheng, Feiyue Ma, Changhui Ge, Hanjiang Fu, Yu Sun, Longteng Wang, Junting Wang, Xuemei Bai, Hao Li, Xiaochen Bo, Hebing Chen, Xiaofei Zheng

**Affiliations:** ^1^ Academy of Military Medical Sciences Beijing China; ^2^ Center for Statistical Science Center for Bioinformatics School of Life Sciences Peking University Beijing China

**Keywords:** lung adenocarcinoma, radiation‐associated cell states, radioresponse heterogeneity, single‐cell sequencing, transcriptional and regulatory landscape

## Abstract

Radioresistance severely compromises radiotherapy efficacy for lung adenocarcinoma (LUAD), yet the intrinsic heterogeneity of cellular radioresponses among isogenic tumor cells remains poorly characterized. Here, we integrated single‐cell RNA sequencing (scRNA‐seq) and single‐cell assay for transposase‐accessible chromatin with high‐throughput sequencing (scATAC‐seq) to dissect the transcriptional and regulatory landscape underlying heterogeneous radioresponses in single‐cell‐derived LUAD cells following 6 Gy γ‐ray irradiation. We found that isogenic LUAD cells harbored intrinsic molecular determinants for divergent radiosensitivity and displayed heterogeneous radioresponsiveness upon irradiation. A radiation‐associated transcriptional cell state, coupled with DNA damage repair, apoptosis, and cell cycle regulation, emerged at 6 h post‐irradiation. Inhibition of lysine demethylase 4B (*KDM4*B) enhanced LUAD radiosensitivity by intensifying DNA damage, whereas reduced expression of cyclin B1 (*CCNB1*) and DNA topoisomerase IIα (*TOP2A*) potentially contributed to radiation‐triggered G2/M arrest. Notably, the radiation‐associated regulatory cell state emerged earlier than radiation‐induced transcriptional cell state. RNA polymerase II subunit A (POLR2A) functioned as a core regulator in high‐responsive LUAD cells. Based on these findings, we proposed a hypothesis for the radiosensitization of LUAD cells. Collectively, this study provides multilayer mechanistic insights into LUAD radioresponse heterogeneity and yields actionable molecular candidates for developing improved radiosensitization strategies to advance precise radiotherapy for LUAD.

## Graphical abstract

Radiation‐associated transcriptional and regulatory cell states drive heterogeneous radioresponses in isogenic lung adenocarcinoma cells.

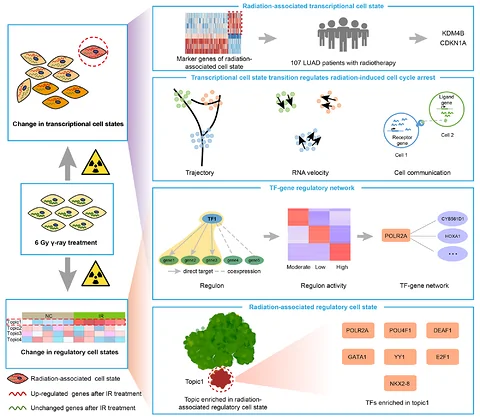

## 1 Introduction

Radiotherapy is widely used to eliminate cancer cells or reduce tumor size before surgery [1]. Its therapeutic effect relies in part on the genomic instability present in various types of cancer [2]. By damaging DNA, radiation can either kill cancer cells or slow down their growth [3]. Once the DNA damage becomes irreparable, the cancer cells are broken down and eliminated [3]. However, radiotherapy efficacy can be compromised by biological changes in cancer cells and their surrounding environment, such as altered DNA damage response (DDR) and cell cycle modifications [4]. Thus, elucidating the mechanisms governing cellular responses to radiation is essential for improving radiotherapy outcomes.

Radiation resistance is a major cause of cancer therapeutic failure, recurrence, and metastasis, specifically in cases like lung adenocarcinoma (LUAD), the most common histologic subtype of lung cancer [5, 6]. Recent research has shown that radiosensitivity varies considerably among patients, tumors and even isogenic cancer cells [7, 8, 9, 10]. This heterogeneity stems from differences in intrinsic radiosensitivity and the complex mechanisms governing cellular radiation responses [11]. The fate of irradiated cells is governed by the extent of radiation‐induced DNA damage and the capacity for early repair [12]. Specifically, molecular factors that regulate the degree of radiation‐mediated DNA damage and the early DNA repair capacity of cancer cells ultimately determine cellular radiosensitivity or radioresistance. Although radioresponse heterogeneity has become a central issue in cancer therapy, its specific molecular mechanisms remain incompletely understood.

A major challenge is identifying cancer cells with different levels of radiosensitivity. Conventional approaches for assessing cellular radioresponse include immunofluorescence staining for evaluating DNA damage and flow cytometry to evaluate radiation‐induced cycle arrest and apoptosis [13]. However, it is difficult to simultaneously verify the radiosensitivity of cancer cells and characterize their biological effects using these traditional experimental methods where only one or several genes can be validated at the same time. This is where single‐cell sequencing comes into the picture [14]. Since its introduction in 2009, single‐cell sequencing has rapidly advanced our insights into embryonic development, neurobiology, immunology, tumor heterogeneity, and personalized therapy [15, 16, 17, 18, 19, 20]. Despite its broad applications in biomedical research, single‐cell sequencing has not been fully exploited to dissect radioresponse heterogeneity among cancer cells. We therefore performed single‐cell RNA sequencing (scRNA‐seq) and single‐cell assay for transposase‐accessible chromatin with high‐throughput sequencing (scATAC‐seq) to characterize the transcriptional and regulatory landscape of irradiated LUAD cells and clarify the molecular mechanisms underlying cellular radioresponse heterogeneity.

In this study, we performed scRNA‐seq and scATAC‐seq of LUAD cells after 6 Gy γ‐ray treatment to dissect their radioresponse heterogeneity from the perspective of transcriptional and regulatory landscape (Figure 1). Based on the molecular features associated with heterogeneous radioresponsiveness in LUAD cells, we further proposed a mechanistic hypothesis and candidate potential targets for radiosensitizing LUAD cells (Figure 1).

**FIGURE 1:**
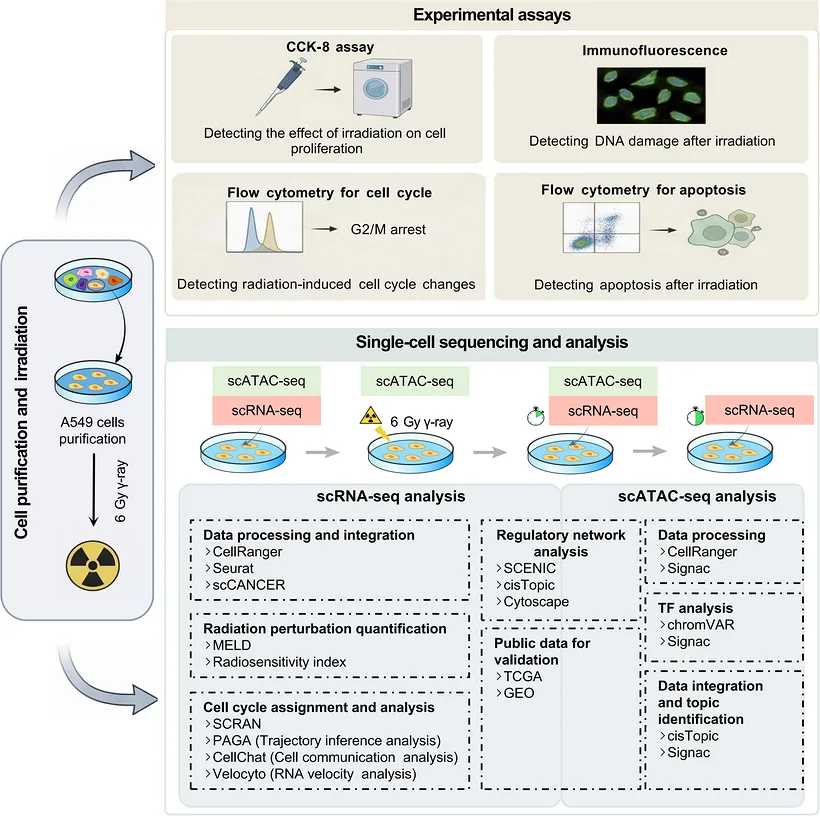
Schematic illustration of the experimental design and data‐analysis workflow. CCK‐8, Cell Counting Kit‐8; GEO, Gene Expression Omnibus; PAGA, partition‐based graph abstraction; SCENIC, single‐cell regulatory network inference and clustering; TCGA, The Cancer Genome Atlas.

## 2 Results

### 2.1 Isogenic LUAD Cells Possess a Molecular Basis for Heterogeneous Radiosensitivity

Recent studies have shown that therapy‐resistant fates can be predetermined by the intrinsic differences in the molecular states of cancer cells before drug exposure [9, 21]. However, whether isogenic cancer cells harbor pre‐existing transcriptional and regulatory heterogeneity that leads to the heterogeneous radioresponses of LUAD cells remains unclear. Therefore, we monoclonally cultured A549 cells, a widely authenticated model of LUAD, and performed scRNA‐seq and scATAC‐seq to characterize their transcriptomic and chromatin‐accessibility landscape (Figure 2A).

**FIGURE 2:**
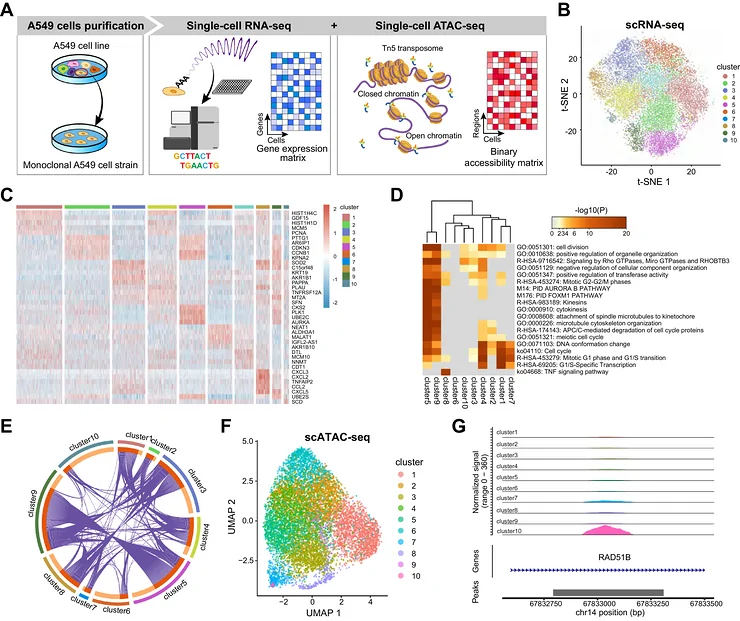
Transcriptomic and chromatin‐accessibility landscape of lung adenocarcinoma (LUAD) cells. (A) Schematic of single‐cell RNA sequencing (scRNA‐seq) and single‐cell assay for transposase‐accessible chromatin with high‐throughput sequencing (scATAC‐seq) of single‐cell‐derived LUAD cells. (B) A t‐distributed stochastic neighbor embedding (t‐SNE) view of 13,619 LUAD cells based on scRNA‐seq data, colored by assigned cell cluster. (C) Heatmap of the average scaled expression of marker genes in each cell cluster. (D) Heatmap of the top functional‐enrichment terms in each cluster, colored by *p* value. The *p* values were calculated using the cumulative hypergeometric distribution and terms with *p* < 0.01 were collected. “log10 (P)” is the *p* value in log base 10. (E) Circos plot showing marker genes shared among clusters. (F) Uniform manifold approximation and projection (UMAP) representation of single‐cell‐derived LUAD cells based on scATAC‐seq data, colored by assigned cell cluster. (G) Normalized chromatin accessibility (coverage plot) of RAD51 paralog B (*RAD51B*) in each cluster.

First, we obtained transcriptomic profiles for 13,619 LUAD cells (Table S1, Figure S1, Section 5) and identified 10 cell clusters using scCANCER [22], a data‐driven machine learning algorithm for automated analysis of cancer scRNA‐seq data (Figure 2B, Supporting Information Methods). Cells in different clusters exhibited diverse transcriptional landscapes (Figure 2C). Gene enrichment analysis showed that the transcriptional states of single‐cell‐derived LUAD cells were mainly associated with cell cycle–related processes, including cell cycle progression and DNA conformation changes (Figure 2D, Supporting Information Methods). Some clusters showed functional similarities, such as cluster 5 and cluster 9, whose marker genes largely overlapped (Figure 2D,E). Cluster 10 showed little functional similarity to the other clusters (Figure 2D), and protein–protein interaction analysis indicated that cells in cluster 10 were active in adenosine triphosphate (ATP) synthesis (Figure S2). ATP has been linked to cancer resistance by promoting enhanced DNA repair [23]. Thus, cluster 10 may possess molecular features associated with radioresistance.

We next examined whether single‐cell‐derived LUAD cells also differed in their regulatory properties. After basic quality control, we obtained chromatin‐accessibility profiles for 8927 cells (Table S2, Figure S3A–D, Supporting Information Methods) and identified 10 cell clusters with different chromatin‐accessibility patterns from the scATAC‐seq data using Signac (Figure 2F, Supporting Information Methods) [24]. Relative chromatin accessibility revealed heterogeneous gene activity, including that of RAD51 paralog B (*RAD51B*), a key recombinase for remodeling and protecting DNA replication fork (Table S3, Figure 2G) [25, 26]. These results demonstrated that LUAD cells exhibited heterogeneous regulatory states.

Above all, even single‐cell‐derived LUAD cells exhibit transcriptional and regulatory heterogeneity associated primarily with cell cycle progression and changes in genome conformation. Cell cycle states and genome architecture are well‐established intrinsic regulators of cellular radiosensitivity and radioresistance [4, 27]. Cells are most radiosensitive in G2/M phase and least radiosensitive in S phase [28], while transcriptionally active chromatin compartments are more susceptible to radiation than transcriptionally repressed compartments [27]. Three‐dimensional (3D) chromatin architectures change dynamically with cell cycle progression and reciprocally regulate gene expression. In turn, 3D genome organization influences radiation‐induced DNA damage and repair [29]. These observations suggest that targeting genes involved in cell cycle regulation and chromatin organization before irradiation may provide a strategy for modifying the intrinsic radiosensitivity of cancer cells.

### 2.2 Transcriptional Changes Reveal Heterogeneous Radiation Responses in Irradiated LUAD Cells

#### 2.2.1 Quantifying the Radiation Response of Irradiated LUAD Cells Using scRNA‐seq Data

We questioned whether single‐cell‐derived LUAD cells respond heterogeneously during the early period after irradiation. Therefore, we irradiated single‐cell‐derived LUAD cells with 6 Gy γ‐ray and performed scRNA‐seq at 2 h and 6 h post‐irradiation (Figure 3A). After quality control, 15,270 and 13,958 cells passed the filtering criteria at the two time points, respectively (Table S1, Figure S1, Supporting Information Methods). To quantify cellular radiation responses, we applied MELD, an algorithm measuring the effect of experimental perturbation from scRNA‐seq data (Figure 3A, Supporting Information Methods) [30]. MELD represents transcriptomic space as a manifold and estimates sample‐associated densities across the manifold of cell states (Figure 3A) [30]. For each cell, a relative likelihood ranging from 0 to 1 is then calculated to quantify the effect of γ‐ray exposure (Figure 3A, Supporting Information Methods). In short, MELD quantifies the transcriptional change of each irradiated LUAD cell and expresses it as an ionizing radiation (IR)‐associated likelihood. Based on the likelihood, irradiated cells were classified into high‐, moderate‐, and low‐responsive groups (Figure 3B, Supporting Information Methods). Moderate‐responsive cells predominated at 2 h post‐irradiation, whereas high‐responsive cells constituted the largest proportion at 6 h (Figure 3B).

**FIGURE 3:**
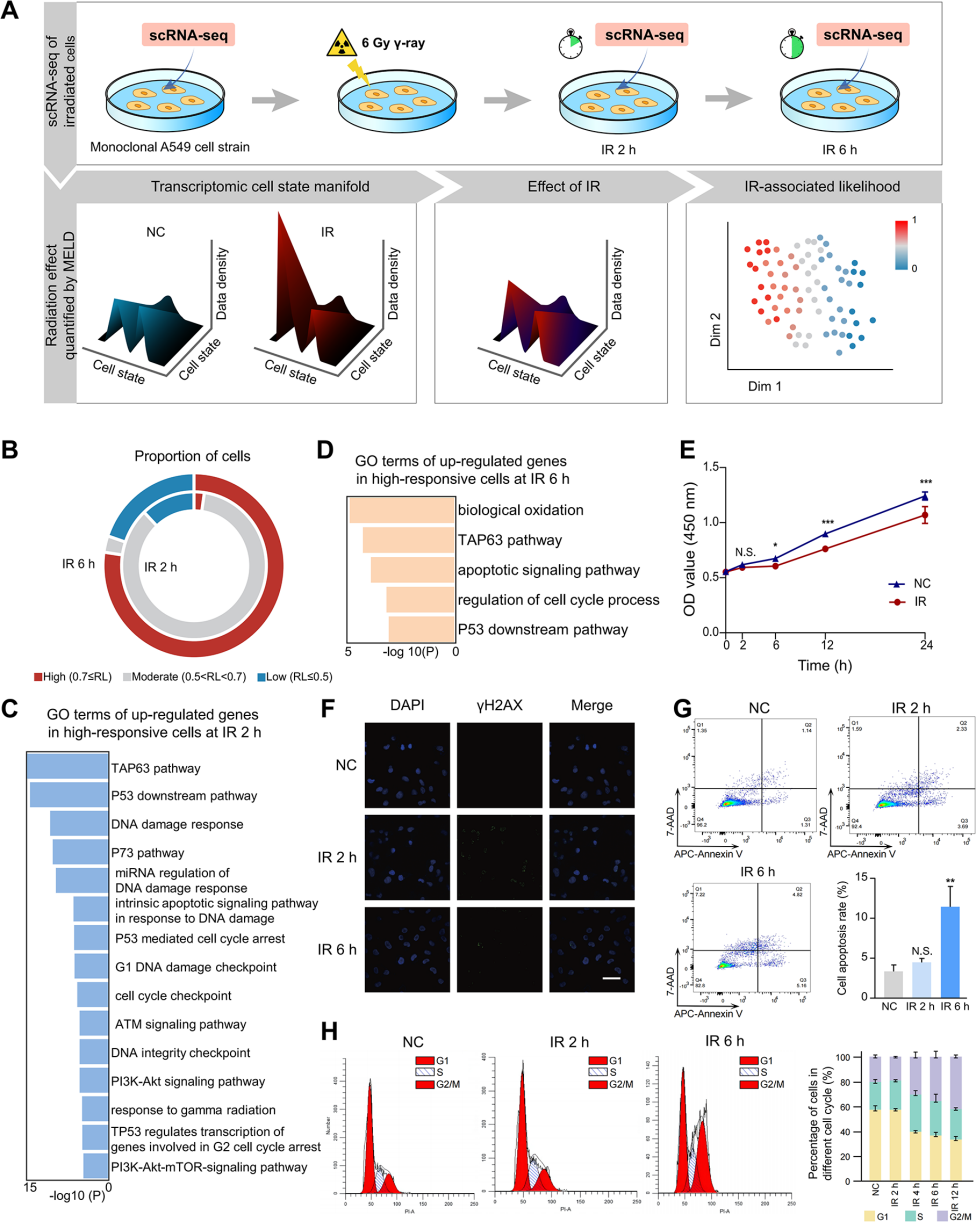
Quantifying cellular radiation responses using scRNA‐seq data. (A) Schematic illustration of scRNA‐seq analysis and quantification of radiation effects using MELD. (B) Proportion of cells with different radiation responses. Irradiated LUAD cells were classified as high‐, moderate‐, and low‐responsive based on ionizing radiation (IR)‐associated likelihood calculated by MELD. (C and D) Functional‐enrichment terms for genes upregulated in high‐responsive versus low‐responsive LUAD cells at 2 h (C) and 6 h (D) post‐irradiation. *p* values were calculated using the cumulative hypergeometric distribution, and terms with *p* < 0.01 were retained. “log10 (P)” is the *p* value in log base 10. (E) Cell Counting Kit‐8 (CCK‐8) assay of proliferation after irradiation. Optical‐density (OD) values are shown as mean ± standard deviation (SD) from five replicates (*n* = 5). Differences among groups were assessed by one‐way analysis of variance (ANOVA) followed by Tukey's post hoc test. Significance markers were defined as follows: not significant (N.S.), *p* > 0.05; *p < 0.05; **p < 0.01; ***p < 0.001. (F) Phosphorylated histone H2AX (γH2AX) immunofluorescence in LUAD cells at different times after 6 Gy γ‐ray irradiation. Scale bar = 50 µm. (G and H) Flow‐cytometric analysis using 7‐aminoactinomycin D (7‐AAD)‐Annexin V staining (G) and propidium iodide staining (H) to evaluate apoptosis and cell cycle distribution, respectively. Values in (G) and (H) are shown as mean ± SD from at least three replicates. Statistical significance in (G) was assessed by a two‐tailed Student's *t*‐test using the significance thresholds defined above.

We next explored the responses of LUAD cells at each time upon γ‐ray exposure. Genes highly expressed in high‐responsive cells relative to low‐responsive cells were identified at each time point. Functional‐enrichment analysis suggested that DNA damage related processes were prominent in high‐responsive cells at 2 h post‐irradiation, whereas apoptosis and cell cycle regulation were more active at 6 h (Figure 3C,D, Supporting Information Methods). We then investigated the biological effects of 6 Gy γ‐ray on cell proliferation, DNA damage, cell cycle distribution, and apoptosis in LUAD cells at both time points (Section 5). Proliferation was reduced at 6 h after irradiation, whereas no significant difference was observed at 2 h (Figure 3E). DNA damage in irradiated LUAD cells was assessed by immunofluorescence measurement of phosphorylated histone H2AX (γH2AX) signals (Section 5). γH2AX signals were more obvious at 2 h than at 6 h post‐irradiation, and DNA damage was unevenly distributed among irradiated LUAD cells (Figure 3F, Figure S4A). These findings suggest that the high‐responsive population at 2 h included LUAD cells with more severe DNA damage (Figure 3C,F). Flow cytometry analysis showed that irradiated LUAD cells were apoptotic at 6 h, but not at 2 h (Figure 3G, Section 5). Similarly, significant G2/M arrest was observed at 6 h, whereas no obvious cell cycle change was detected at 2 h (Figure 3H, Section 5). These experimental findings were aligned with the scRNA‐seq analyses and supported classification of LUAD cells according to IR‐associated likelihood (Figure 3C,D).

#### 2.2.2 A Radiation‐Associated Transcriptional Cell State Emerges in LUAD Cells at 6 h Post‐Irradiation

We next tested if there were radiation‐associated transcriptional cell states in irradiated LUAD cells that newly appeared or disappeared post‐irradiation. We integrated scRNA‐seq data of irradiated and non‐irradiated LUAD cells by identifying their shared anchors using Seurat (Figure 4A, Supporting Information Methods) [22, 31]. No newly appeared transcriptional state was detected at 2 h post‐irradiation (Figure 4B, Figure S4B), whereas a radiation‐associated transcriptional cell state emerged at 6 h post‐irradiation. Cells in this state were the most strongly affected by radiation according to MELD (Figure 4B, Figure S4C).

**FIGURE 4:**
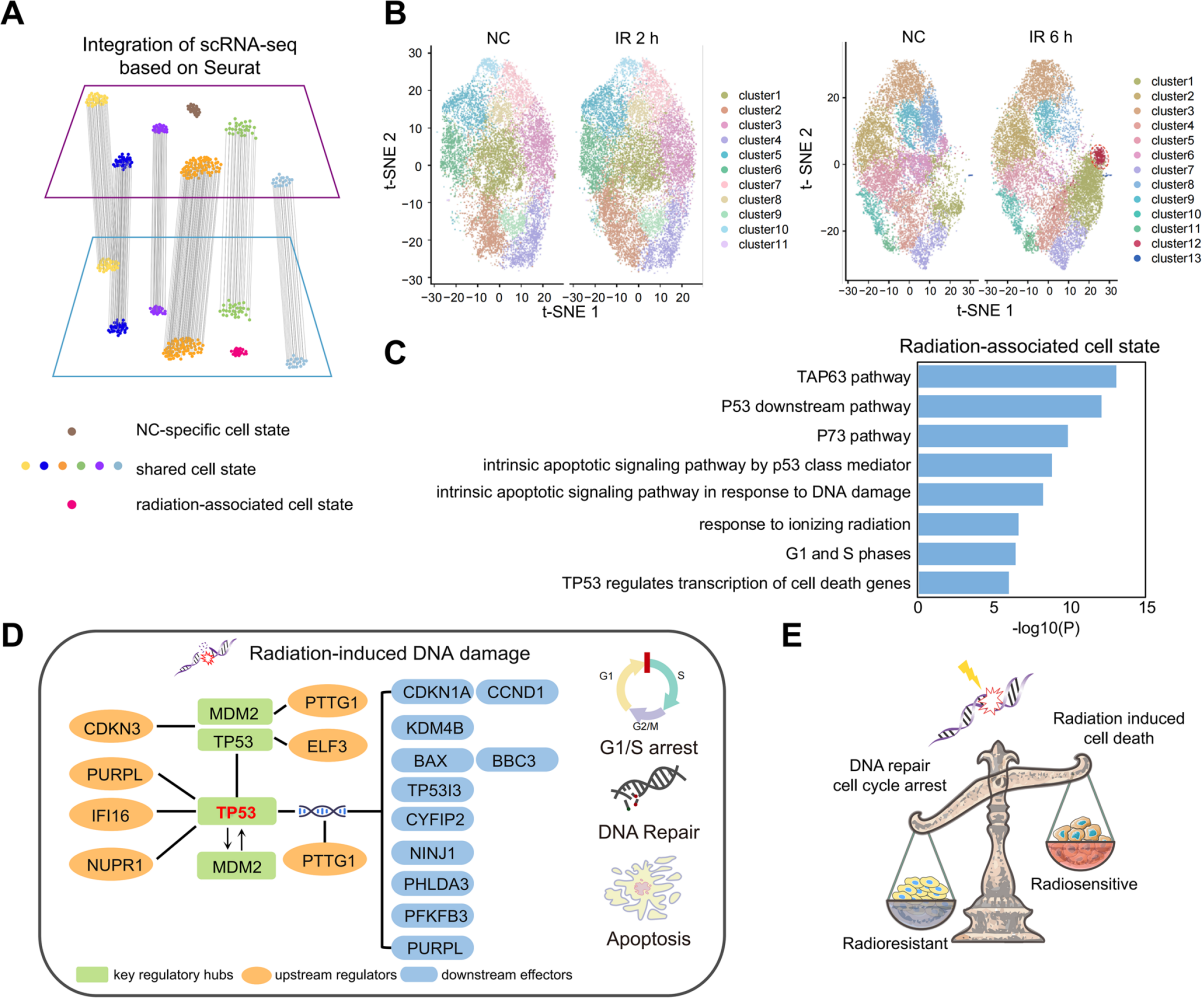
Radiation‐associated transcriptional cell state. (A) Integrating scRNA‐seq data of control sample and irradiated LUAD cells to identify radiation‐associated transcriptional cell state. (B) t‐SNE view of integrated scRNA‐seq data, colored by re‐evaluated clusters. Red circles indicate the radiation‐associated transcriptional cell state at 6 h post‐irradiation; data from each post‐irradiation time point were integrated separately with the control data. (C) Gene Ontology (GO) analysis of key genes in the radiation‐associated cell state. *p* values were calculated using the cumulative hypergeometric distribution, and terms with *p* < 0.01 were retained. “log10 (P)” is the *p* value in log base 10. (D) Tumor protein p53 (*TP53*) signaling pathway in the radiation‐associated transcriptional state. Genes shown in the pathway were marker genes of this state. (E) Schematic illustration of cell‐fate decisions in irradiated LUAD cells.

Genes highly expressed in the radiation‐associated cell state were enriched for tumor protein p53 (*TP53*)‐mediated DNA repair, apoptosis, and cell cycle regulation (Figure 4C,D, Table S4). The cell cycle assignment using SCRAN showed that radiation‐associated transcriptional cell state mainly contained cells in G1/S phase (Figure S4D, Supporting Information Methods) [32]. Accordingly, the state was enriched for irradiated G1/S‐phase LUAD cells with irreparable DNA damage (Figure 4C,D, Figure S4D). We then identified differentially expressed genes between the radiation‐associated transcriptional cell state and LUAD cells with relatively low responsiveness (cluster 5) (Figure S4C,E). Genes highly expressed in the relatively low‐responsive cells were enriched for radiation‐induced DNA damage repair through the ataxia telangiectasia and RAD3‐related (*ATR*) signaling pathway (Figure S4F). These results suggest a complex regulatory process at 6 h post‐irradiation in which the balance between DNA damage and repair is a key determinant of cell fate (Figure 4E). When DNA damage is severe and irreparable, cells will undergo apoptosis to protect genomic integrity in their progeny [33, 34].

#### 2.2.3 Key Genes in the Radiation‐Associated Transcriptional Cell State

To identify key genes in the radiation‐associated transcriptional cell state that may contribute to LUAD radiosensitivity, we reconstructed the *TP53* signaling pathway using genes highly expressed in this state (Figure 4D). The pathway contained multiple *TP53*‐related effectors and regulators involved in cell cycle arrest, DNA repair, and apoptosis (Figure 4D) [35, 36]. To examine their relevance in patients receiving radiotherapy, we analyzed clinical data from 107 LUAD patients in The Cancer Genome Atlas (TCGA) database (Tables S5 and S6, Supporting Information Methods). Radiosensitivity was estimated using the radiosensitivity index (RSI), a multi‐gene expression index that is negatively correlated with intrinsic tumor radiosensitivity (Supporting Information Methods) [37]. Patients were classified as radioresistant or radiosensitive using the median RSI as the cutoff: RSI ≥ 0.225 indicated radioresistance, whereas RSI < 0.225 indicated radiosensitivity. Based on the definition of radiosensitivity, we found that lysine demethylase 4B (*KDM4B*) and cyclin‐dependent kinase inhibitor 1A (*CDKN1A*) were upregulated in radioresistant cases (Figure 5A). *KDM4B* encodes a histone lysine demethylase involved in cell cycle regulation, DNA damage repair, and cellular stress responses [38]. We therefore assessed cell proliferation and DNA damage in LUAD cells at 6 h after γ‐ray irradiation following *KDM4B* inhibition with NCGC00244536 (Section 5). *KDM4B* inhibition increased cellular sensitivity to 6 Gy γ‐radiation and exacerbated DNA damage in LUAD at 6 h post‐irradiation (Figure 5B,C), supporting *KDM4B* as a promising therapeutic target for enhancing LUAD radiosensitivity. Previous studies have shown that *CDKN1A* can reduce cellular radiosensitivity by promoting DNA repair and G1/S arrest, suggesting that it may also represent a potential radiosensitization target in LUAD [39, 40].

**FIGURE 5:**
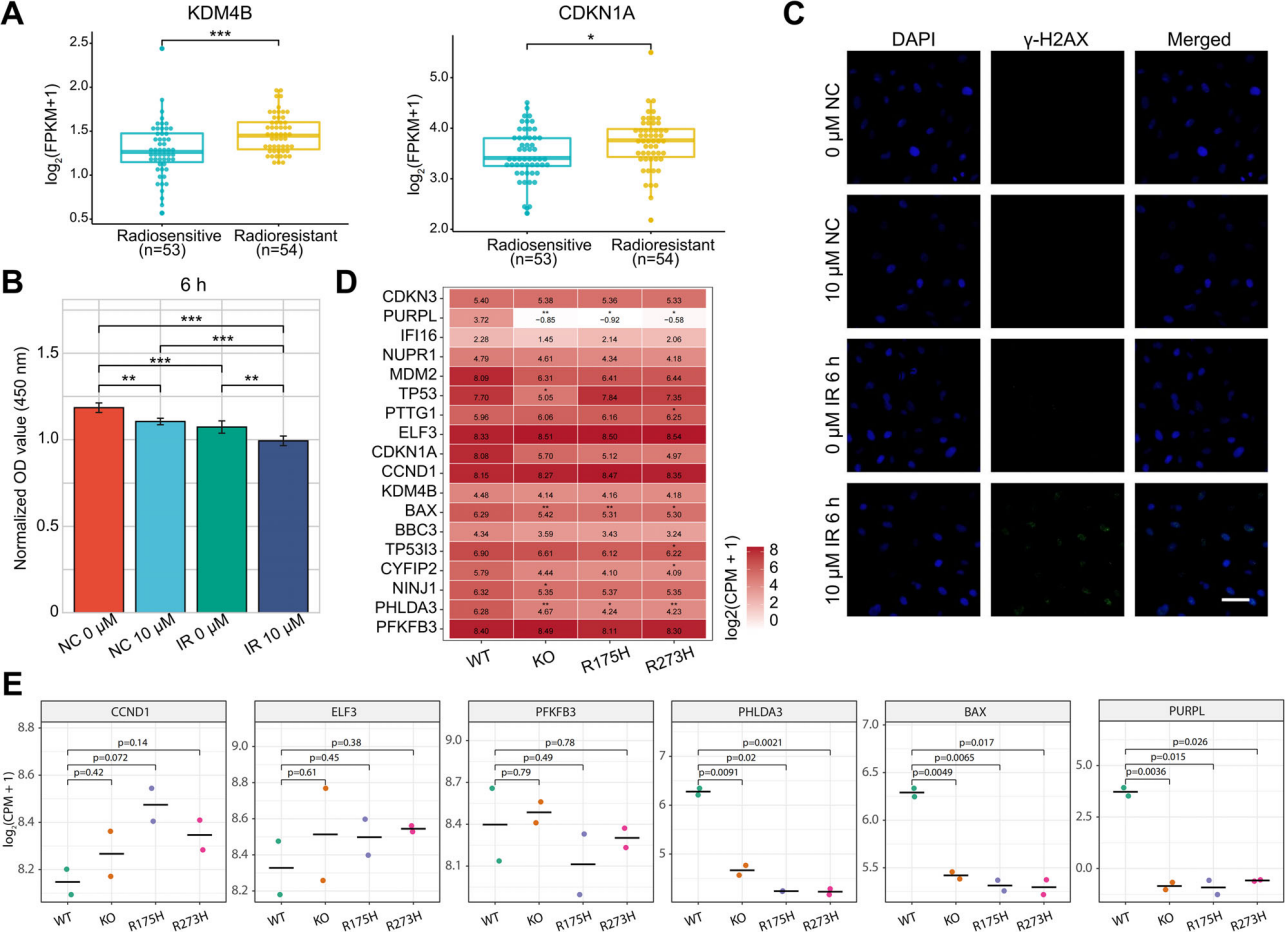
Lysine demethylase 4B (*KDM4B*) inhibition enhances radiosensitivity in LUAD cells and key radiation‐response genes show differential expression according to *TP53* status. (A) *KDM4B* (left) and cyclin‐dependent kinase inhibitor 1A (*CDKN1A*) (right) expression in radiosensitive versus radioresistant patients, color‐coded by radiosensitivity. Radiosensitivity was defined using the radiosensitivity index (RSI) in 107 LUAD patients who received radiotherapy in The Cancer Genome Atlas (TCGA) cohort. Statistical significance was assessed by a two‐tailed Student's *t*‐test. Significance markers were defined as follows: not significant (N.S.), *p* > 0.05; *p < 0.05; **p < 0.01; ***p < 0.001. (B) CCK‐8 assay was used to detect the proliferative capacity of LUAD cells with *KDM4B* inhibition at 6 h post‐irradiation. OD values are presented as mean ± SD (*n* = 5). One‐way ANOVA combined with Tukey's post hoc test was adopted for multi‐group and pairwise comparisons. Statistical significance was defined as *p* < 0.05; statistical markers are consistent with panel (A). (C) Immunofluorescence staining of γH2AX was performed in *KDM4B*‐inhibited LUAD cells at 6 h post‐irradiation. Scale bar = 50 µm. The four experimental groups in (B) and (C) were negative controls treated with 0 or 10 µM NCGC00244536 and ionizing radiation (IR) groups treated with 0 or 10 µM NCGC00244536. (D) Heatmap of average scaled expression of core radiation‐response genes in A549 cells with wild‐type *TP53*, *TP53* knockout, or *TP53* R175H/R273H mutations. Values are colored by log2[counts per million (CPM) + 1]. (E) Bar plot of average scaled expression of cyclin D1 (*CCND1*), E74‐like ETS transcription factor 3 (*ELF3*), 6‐phosphofructo‐2‐kinase/fructose‐2,6‐bisphosphatase 3 (*PFKFB3*), pleckstrin homology‐like domain family A member 3 (*PHLDA3*), BCL2‐associated X protein (*BAX*), and p53 upregulated regulator of p53 levels (*PURPL*). Black bars indicate the mean of two replicates (*n* = 2). Statistical significance was assessed by a two‐tailed Student's *t*‐test; *p* < 0.05 was considered significant, with annotations as defined in (A).

*TP53*‐related genes critically regulate the radiation response of LUAD cells, yet *TP53* mutations are highly prevalent in LUAD patients [41]. To investigate how *TP53* deficiency affects the cellular response to radiation, we analyzed Gene Expression Omnibus (GEO) datasets to determine how *TP53* knockout and mutation altered the expression of key genes in the radiation‐associated transcriptional cell state (Figure 5D, Supporting Information Methods). Expression of several genes, including cyclin D1 (*CCND1*), E74‐like ETS transcription factor 3 (*ELF3*), and 6‐phosphofructo‐2‐kinase/fructose‐2,6‐bisphosphatase 3 (*PFKFB3*), did not depend on *TP53* status (Figure 5E). In contrast, pleckstrin homology‐like domain family A member 3 (*PHLDA3*), BCL2‐associated X protein (*BAX*), and p53 upregulated regulator of p53 levels (*PURPL*) were downregulated under both *TP53‐knockout* and *TP53*‐mutant conditions (Figure 5E). *PHLDA3* and *BAX* participate in *TP53*‐mediated apoptosis [42, 43, 44]. *PURPL* is a nuclear long noncoding RNA directly activated transcriptionally by wild‐type *TP53* after DNA damage or genomic stress and forms a negative auto‐regulatory feedback loop with *TP53* [45]. Collectively, *TP53* status does not determine the expression of all key radiation‐response genes, but it substantially modulates the radiotherapeutic response of LUAD cells. The *TP53* genetic background should therefore be considered when screening and validating radiosensitizing targets for LUAD treatment.

### 2.3 Characteristic Transcriptional Changes Accompanied With Radiation‐Induced Cell Cycle Arrest

#### 2.3.1 Key Genes for Cell Cycle Transition Are Reduced in G2/M Arrested LUAD Cells

We further explored how transcriptional changes in irradiated LUAD cells were associated with radiation‐induced cell cycle arrest. We performed trajectory inference, RNA velocity, and cell communication analysis using scRNA‐seq data (Figure 6A, Supporting Information Methods). We annotated the cell cycle phases of irradiated and non‐irradiated LUAD cells. A pronounced G2/M arrest at 6 h after irradiation was consistent with the cell cycle profiles detected by flow cytometry (Figure 6B). Trajectory analysis using partition‐based graph abstraction (PAGA)‐tree [46] showed that the transcriptional changes were closely associated with G2/M arrest at 6 h post‐irradiation (Figure 6C, Supporting Information Methods). Cyclin B1 (*CCNB1*) and DNA topoisomerase IIα (*TOP2A*) were downregulated in G2/M arrested LUAD cells (Figure 6C,D). This finding is consistent with previous reports that reduced *CCNB1* expression and impaired *TOP2A* function are closely linked to G2/M arrest [47, 48]. Nevertheless, the exact relationship between downregulation of these two genes and radiation‐induced G2/M arrest in LUAD cells requires further elucidation.

**FIGURE 6:**
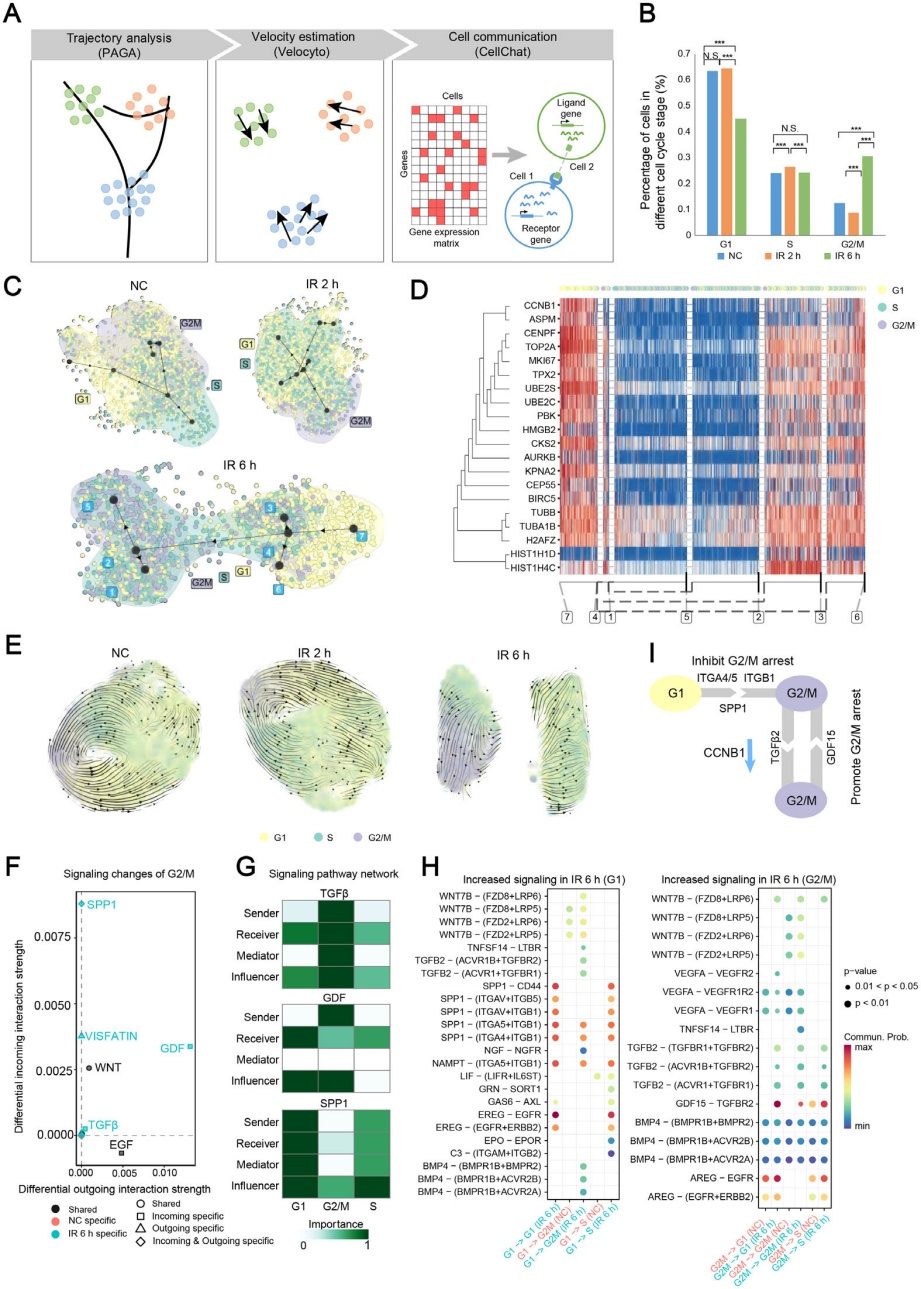
Characteristic transcriptional changes associated with radiation‐induced cell cycle arrest. (A) Schematic diagram of cell cycle analysis using scRNA‐seq data, including trajectory inference, RNA velocity and cellular communication. (B) Proportion of cells in different cell‐cyclephases. Cells were assigned into cell cycle phases using SCRAN. The *p* values were calculated using two‐sample *Z*‐test. Significance markers were defined as follows: not significant (N.S.), *p* > 0.05; *p < 0.05; **p < 0.01; ***p < 0.001. (C) Differential trajectory of cell cycle transition in control and irradiated LUAD cells, projected onto a two‐dimensional uniform manifold approximation and projection (UMAP) embedding. Colors indicate cell cycle phases, arrows indicate inferred transition directions, and numbers mark key milestones in the 6 h post‐irradiation trajectory. (D) Heatmap of the gene expression changes associated with cell cycle transition in LUAD cells at 6 h post‐irradiation. The upper bar was colored based on milestones. (E) RNA velocity of LUAD cells, projected on a 2D UMAP embedding, where color represents different cell cycle. (F) Comparison of significant signaling in different cell cycle between irradiated LUAD cells at 6 h post‐irradiation and negative control, where color and shape represent sample and interaction mode, respectively. (G) Heatmaps showing the relative importance of each cell cycle phase based on network‐centrality measures for transforming growth factor beta (*TGFβ*), growth differentiation factor (*GDF*), and secreted phosphoprotein 1 (*SPP1*) signaling. (H) Communication probability of the increased signaling network of each ligand‐receptor pair in LUAD cells of different cell cycles at 6 h post‐irradiation. *p* values were obtained from CellChat permutation tests, and dot size is proportional to the *p*‐value interval. (I) Illustrative description of intercellular communication among irradiated LUAD cells in different cell cycles.

We subsequently analyzed RNA velocity using dynamo (Supporting Information Methods) [49]. RNA velocity assesses both spliced and unspliced mRNA to predict changes in gene expression over time [50]. The analysis indicated that transcriptional transition from G2/M to G1 was substantially altered at 6 h post‐irradiation, but not at 2 h (Figure 6E). Thus, major shifts in the transcriptional landscape coincided with radiation‐induced cell cycle arrest, supporting a close association between radiation‐elicited transcriptional changes and cell cycle progression.

#### 2.3.2 Intercellular Communication Regulates G2/M Arrest in Irradiated LUAD Cells

We next investigated whether intercellular signaling contributed to G2/M arrest in LUAD cells at 6 h post‐irradiation. We performed CellChat, a ligand‐receptor interaction explorer using scRNA‐seq data, to infer intercellular communication networks in LUAD cells (Figure 6A, Supporting Information Methods) [51]. Comparison of G2/M‐phase signaling between irradiated and non‐irradiated cells showed that transforming growth factor beta (*TGFβ*), growth differentiation factor (*GDF*), and secreted phosphoprotein 1 (*SPP1*) specifically mediated incoming signaling in G2/M LUAD cells at 6 h post‐irradiation (Figure 6F). *TGFβ* and *GDF* are members of the transforming growth factor beta superfamily, which includes major cytokines released by cells after radiation exposure [52]. Incoming *TGFβ* and *GDF* signaling in G2/M cells at 6 h post‐irradiation were generated mainly by G2/M cells (Figure 6G), whereas incoming *SPP1* signaling originated primarily from G1 cells (Figure 6G).

We then characterized the ligand–receptor pairs underlying *TGFβ*, *GDF*, and *SPP1* signaling (Figure 6H). Transforming growth factor beta 2 (*TGFβ2*)‐ and growth differentiation factor 15 (*GDF15*)‐related ligand–receptor interactions showed the highest communication probability, with prominent *TGFβ2* signaling in G2/M‐arrested LUAD cells (Figure 6H). Previous studies suggest that *TGFβ* signaling can downregulate the expression of several G2‐checkpoint kinases including *CCNB1* [53]. In our analyses, increased *TGFβ2*‐related signaling coincided with reduced *CCNB1* expression and G2/M arrest, suggesting a potential regulatory association. Interaction between integrin subunit beta 1 (*ITGB1*) and integrin subunit alpha 4/5 (*ITGA4/5*) regulated the *SPP1* signaling in G2/M LUAD cells at 6 h post‐irradiation (Figure 6H). Previous evidence has indicated that *SPP1* deficiency exacerbates radiation‐evoked G2/M arrest [54], whereas *ITGB1* ablation potentiates this phenotype [55]. These findings suggest that increased *SPP1* signaling may attenuate G2/M arrest. Collectively, *TGFβ2* and *SPP1* signaling may exert opposing effects on radiation‐induced G2/M arrest (Figure 6I). Because pronounced G2/M arrest is associated with cellular radioresistance [56], targeting these signaling regulators may represent potential targets for LUAD radiotherapy, although the underlying mechanisms require further validation.

### 2.4 Radiation‐Associated Regulatory Cell State Emerges Earlier Than Transcriptional Cell State

#### 2.4.1 Characteristic Regulatory Changes in Irradiated LUAD Cells

Radiation‐induced transcriptional changes were not obvious in LUAD cells at 2 h post‐irradiation. Considering that the effects of radiation on DNA should precede changes in gene expression, we questioned whether radiation‐induced chromatin accessibility change could be detected earlier in irradiated LUAD cells. We performed scATAC‐seq in LUAD cells immediately and at 2 h after 6 Gy γ‐ray treatment (Figure 7A). After quality control, 8927, 8596, and 4073 cells from three samples were retained for downstream analysis (Table S2, Figure S3A–D, Supporting Information Methods). Pseudo‐bulk analysis of scATAC‐seq performed by Signac [24] and chromVAR [57] showed that several transcription factors (TFs), including TEA domain (TEAD) and fos‐like antigen (FOSL), exhibited radiation‐associated changes in chromatin accessibility (Figure S5A, Supporting Information Methods). Recent studies have reported that DNA double‐strand breaks (DSBs) frequently occur near TEAD motifs across multiple cell types [58]. Moreover, motifs that lost accessibility after irradiation showed preferred 5′‐C*C or 5′‐CC* dinucleotide sequences (Figure S5B), consistent with the reported prevalence of 5′‐GG* and 5′‐G*G at radiation‐induced DNA‐damage sites [59]. These findings suggest a potential role for these TFs in genome fragility.

**FIGURE 7:**
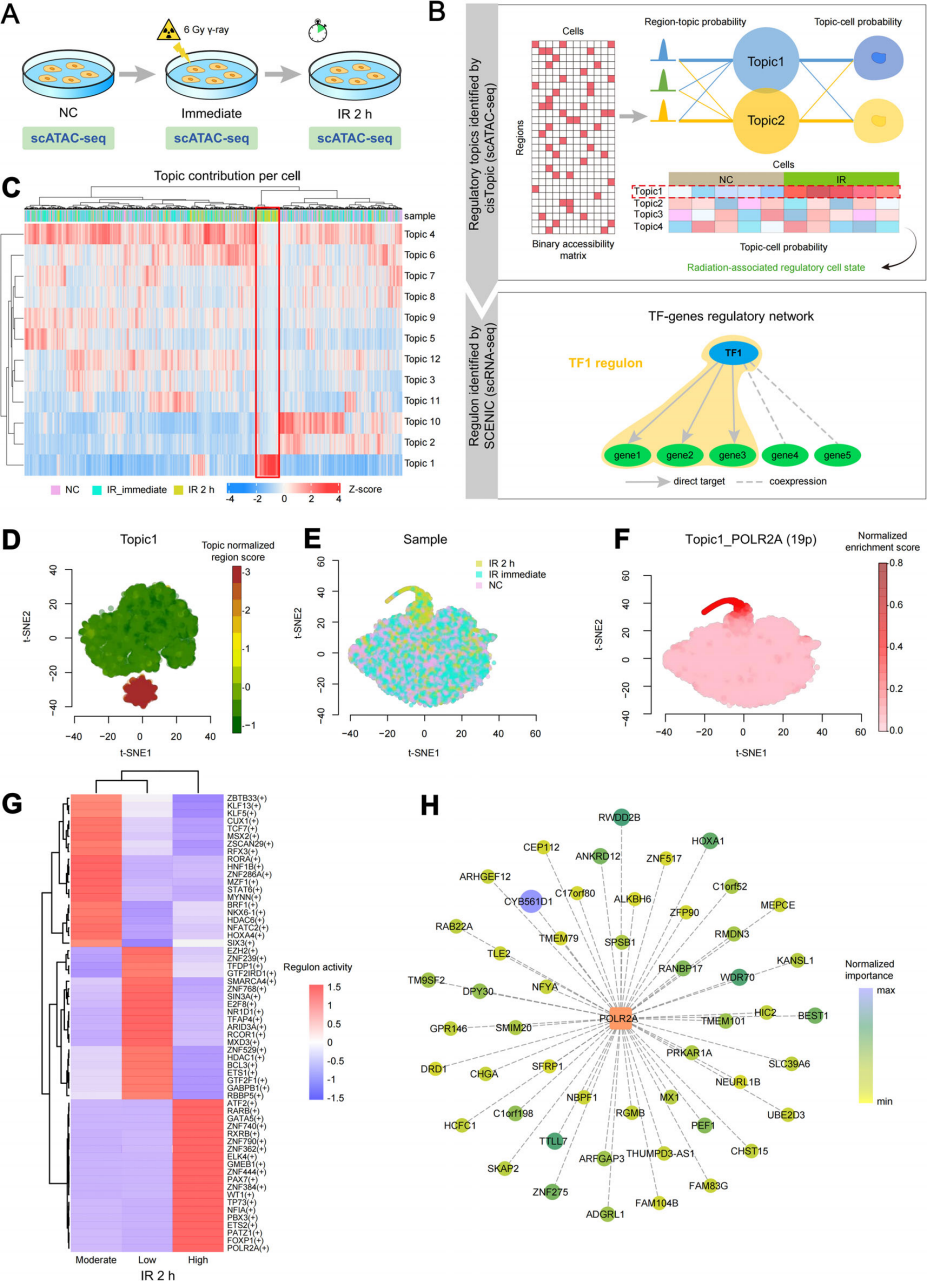
Radiation‐associated regulatory cell state. (A) To quantify the effect of γ‐ray radiation on chromatin accessibility of LUAD cells, LUAD cells were exposed to 6 Gy γ‐ray. Then, scATAC‐seq was performed in non‐irradiated (negative control, NC) and irradiated (immediate and 2 h post‐irradiation) LUAD cells. (B) Schematic illustration of regulatory network analysis. The accessibility matrix was provided as input to cisTopic to estimate the region–topic and topic–cell probability distributions. Single‐cell regulatory network inference and clustering (SCENIC) identifies regulons, defined as transcription factors (TFs) and their predicted target genes from scRNA‐seq data. (C) Heatmap of the topic contribution in each cell, where the contribution was normalized. The red box marked the cells enriched in topic 1. (D) t‐SNE view based on the region–topic distributions colored by the topic normalized region score of topic 1. (E) t‐SNE view based on cell–topic distributions colored by scATAC‐seq samples. (F) Top enriched motifs in topic 1 with normalized enrichment score. t‐SNE view was visualized based on cell–topic distributions. (G) Heatmap of the scaled regulon activity in high‐, moderate‐, and low‐responsive LUAD cells at 2 h post‐irradiation. (H) POLR2A regulatory network in LUAD cells at 2 h post‐irradiation, where circle size and color represent the normalized importance for RNA polymerase II subunit A (POLR2A) regulated genes within the POLR2A regulon.

#### 2.4.2 Radiation‐Associated Regulatory Cell States Are Enriched for Radiation‐Related TFs

To determine whether radiation‐associated regulatory cell states were present, we integrated the three scATAC‐seq datasets and identified regulatory topics of integrated data using cisTopic [60]. cisTopic applies an unsupervised Bayesian topic‐modeling framework to group genomic regions into regulatory topics and estimate the contribution of each topic to individual cells (Figure 7B, Supporting Information Methods) [60]. Twelve topics were identified, and topic 1 contributed specifically to irradiated cells (Figure 7C, Figure S6A). We then performed t‐distributed stochastic neighbor embedding (t‐SNE) using cisTopic wrapper functions and visualized the integrated data according to sample identity and high‐confidence regions (Figure 7D,E, Section 5). Topic 1‐enriched cells separated from the main population, corresponding to the “tail” in the sample‐based t‐SNE plot (Figure 7D,E). To characterize this regulatory state, regions contributing to topic 1 were annotated by genomic region type and TF enrichment using ChIPseeker [61] and RcisTarget [62], respectively. Topic 1 regions were enriched primarily in regulatory regions, including distal intergenic and intronic regions (Figure S6B,C). In addition, RNA polymerase II subunit A (POLR2A), E2F transcription factor 1 (E2F1), Yin Yang 1 transcription factor (YY1), POU class 4 homeobox 1 (POU4F1), DEAF1 transcription factor (DEAF1), GATA binding protein 1 (GATA1), and NK2 homeobox 8 (NKX2‐8) were enriched in topic 1, indicating potential regulatory roles in the cellular radiation response (Figure 7F, Figure S6D).

#### 2.4.3 POLR2A Is a Key TF Regulating Radioresponse in High‐Responsive LUAD Cells

To further characterize the TF‐gene regulatory network in LUAD cells at 2 h post‐irradiation, we performed regulatory‐network analysis using the scRNA‐seq data. Single‐cell regulatory network inference and clustering (SCENIC) infers regulatory relationships between TFs and genes to characterize cell states; a TF together with its predicted target genes is termed a regulon [62]. Using SCENIC, we identified 331 regulons and calculated their activities in LUAD cells at 2 h post‐irradiation (Table S7, Supporting Information Methods). Relative regulon activities were then compared across cells with heterogeneous radiation responses (Figure 7G). Notably, POLR2A, which was enriched in the radiation‐associated regulatory state, was also identified as an active regulon characteristic of high‐responsive cells at 2 h post‐irradiation (Figure 7G,H). POLR2A is a core subunit of RNA polymerase II with an important role in DNA‐damage‐repair signaling [63]. It can mediate an RNA‐dependent DDR by coupling DSB‐induced c‐Abl activity to the generation of damage‐responsive transcripts (DARTs) for subsequent DSB recognition [64]. Our analysis suggested that POLR2A regulated multiple genes in high‐responsive cells at 2 h post‐irradiation, with cytochrome b561 family member D1 (*CYB561D1*) showing the greatest inferred regulatory importance (Figure 7H). *CYB561D1* has a role in ferroptosis regulation, a form of radiation‐induced cancer‐cell death driven by lipid peroxidation [65].

### 2.5 Hypothesis for the Radiosensitization of LUAD Cells

To better elucidate the radiosensitivity of cancer cells, we categorized the cellular radiation response into three sequential stages: the initial stage, the responsive stage, and the fate‐determining stage (Figure 8A). DNA damage serves as the core event throughout this process, and the DDR is pivotal in regulating cellular radiosensitivity.

**FIGURE 8:**
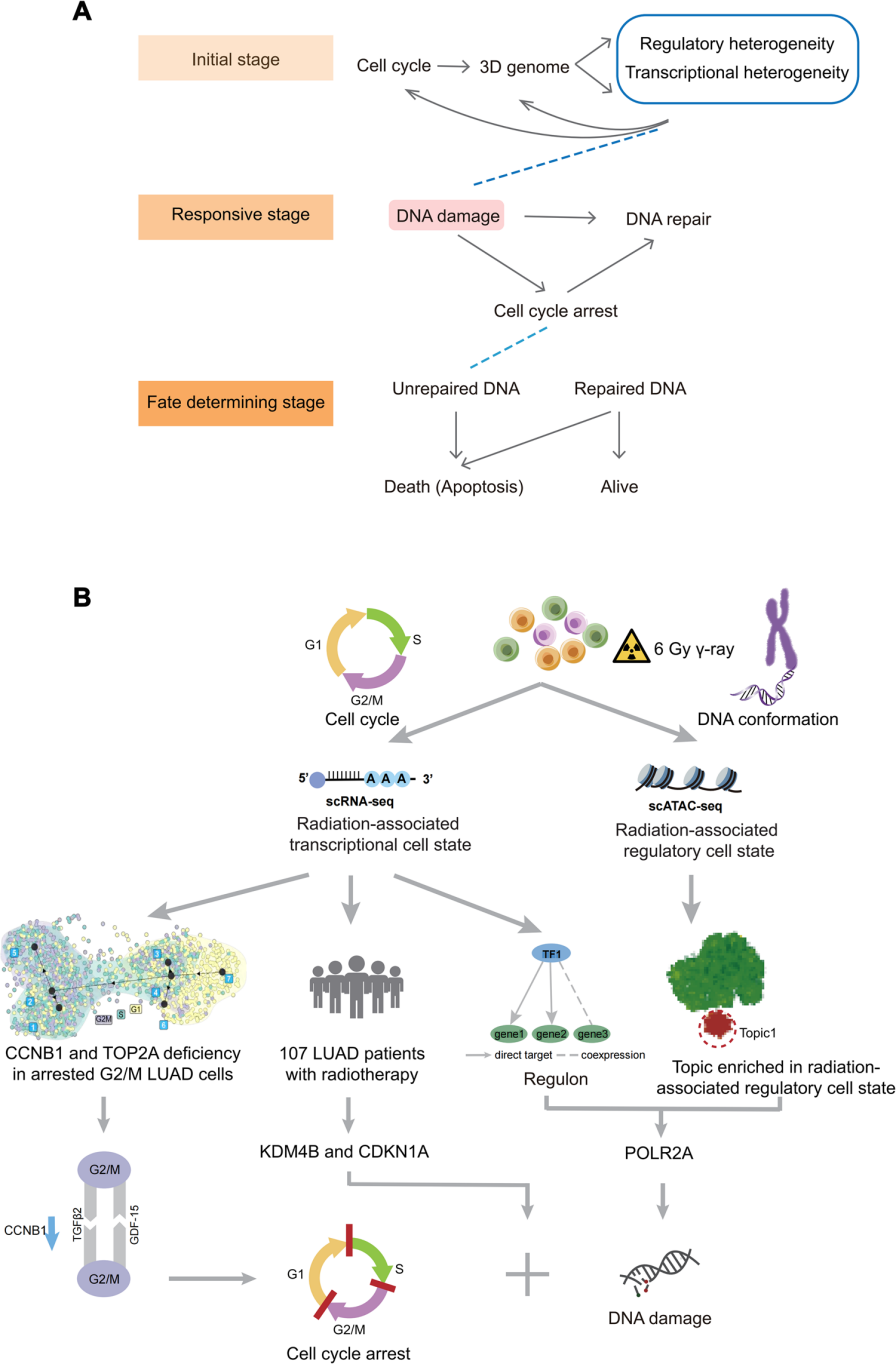
Hypothesis for the radiosensitization of LUAD cells. (A) Three stages of LUAD cells responding to radiation. (B) Proposed mechanism underlying radioresponse heterogeneity of LUAD cells.

At the initial stage, the intrinsic properties of cancer cells are critical determinants of cellular radiosensitivity. Parameters including cell cycle status and chromatin architecture influence DNA‐damage responses following radiation exposure, as reported previously [4, 27]. Here, we found that even non‐irradiated, isogenic LUAD cell populations exhibited transcriptional and regulatory heterogeneity closely associated with cell cycle dynamics and genome conformation (Figure 8B). Genome remodeling driven by cell cycle progression may contribute to this heterogeneity, which in turn may influence cell cycle progression and genome organization. These findings raise the possibility that targeting intrinsic heterogeneity before irradiation could remodel cancer‐cell properties, increase susceptibility to radiation‐induced DNA damage, and ultimately improve radiotherapy efficacy. Further studies are needed to identify molecules that can modulate chromatin structure and cell cycle states without promoting cancer progression.

Cellular responses to radiation‐induced DNA damage are key to radiotherapy efficacy [2]. During the responsive stage, the balance between DNA damage and repair governs cell fate, with DNA repair machinery and cell cycle regulation acting as core determinants (Figure 8A). Radiation‐triggered DNA damage activates cell cycle checkpoints, halting cell cycle progression to avoid division with damaged genome [66]. We observed pronounced G2/M arrest after irradiation, which may facilitate DNA repair (Figure 8B), together with marked reductions in *CCNB1* and *TOP2A* expression (Figure 8B). *TGFβ*‐mediated intercellular communication among G2/M‐arrested LUAD cells may contribute to *CCNB1* downregulation. Modulating *CCNB1* may therefore provide a potential strategy to alleviate G2/M arrest, increase unrepaired DNA damage, and ultimately promote cell death (Figure 8B).

The fate‐determining stage determines whether cancer cells survive or die after irradiation (Figure 8A). At 6 h post‐irradiation, we identified a radiation‐associated transcriptional state in which a small subset of G1/S‐phase cells exhibited substantial DNA damage. These cells showed features of irreparable DNA damage and ultimately underwent apoptosis, consistent with the intended effect of radiotherapy. Characterizing this state identified candidate molecules that may enhance LUAD radiosensitivity (Figure 8B). Our analyses also indicated that radiation‐associated changes emerged earlier at the regulatory level than the transcriptional level. Using scATAC‐seq, we identified a radiation‐associated regulatory state at 2 h post‐irradiation and detected specific motifs potentially involved in the early cellular response to radiation. Integrated scRNA‐seq analysis further identified POLR2A as a core TF in highly responsive LUAD cells. Its inferred regulatory network included ferroptosis‐related and other radiation‐responsive genes, supporting POLR2A as a candidate for further mechanistic investigation.

## 3 Discussion

Radioresistance remains a major obstacle to effective cancer radiotherapy, particularly in the context of precision treatment. Elucidating the molecular drivers of heterogeneous cellular responses to irradiation is therefore essential for developing radiosensitizers and improving therapeutic outcomes. Here, we integrated scRNA‐seq and scATAC‐seq to characterize radioresponse heterogeneity in LUAD cells from transcriptional and regulatory perspectives. Based on these findings, we developed a three‐stage framework for LUAD radioresponse and identified candidate molecular targets for radiosensitization.

A key finding of this work is that LUAD cells exhibit intrinsic transcriptional and regulatory heterogeneity closely coupled to cell cycle dynamics and 3D genome organization. This molecular variation may contribute to divergent radioresponses among otherwise isogenic cancer cells. We also identified radiation‐associated transcriptional and regulatory states, with the regulatory state emerging earlier than the transcriptional state. The radiation‐associated transcriptional state was characterized by activation of *TP53* signaling, a canonical pathway governing DNA‐damage responses and cell death, although not all key genes in this state were *TP53* dependent. Combined analysis of clinical LUAD data and in vitro experiments supported *KDM4B* as a candidate target for enhancing radiosensitivity. In addition, the cell cycle transition genes *CCNB1* and *TOP2A* were reduced in G2/M‐arrested LUAD cells, and *TGFβ2*‐mediated intercellular communication may *contribute* to *CCNB1* downregulation. *POLR2A*, which was enriched in the early radiation‐responsive regulatory state, also emerged as a core regulator in highly responsive LUAD cells.

Integrating these observations, we proposed a three‐stage model that encompasses the initial, responsive, and fate‐determining phases of the LUAD cellular response to irradiation. In addition to the responsive and fate‐determining phases that have been extensively studied, this framework highlights potential intervention opportunities during the initial phase [67, 68]. In particular, modulating chromatin architecture and cell cycle state before irradiation may reshape intrinsic radiosensitivity. Molecules involved in chromatin remodeling, cell cycle control, apoptosis, and DNA‐damage responses, including *KDM4B*, therefore represent candidate targets for LUAD radiosensitization. Further preclinical validation and clinical studies will be required to determine their efficacy, optimize treatment regimens, and maximize therapeutic benefit while minimizing off‐target toxicity.

This study has several limitations. First, all experiments were performed in A549 cells with a fixed *TP53* wild‐type background, which cannot recapitulate the extensive genetic heterogeneity of clinical LUAD, including the high frequency of *TP53* mutations [69, 70]. Genetically engineered cell lines carrying common driver mutations could better model patient‐specific genetic backgrounds and associated radioresponse diversity. Second, conventional two‐dimensional monolayer culture lacks key features of the tumor microenvironment, including hypoxia, immune crosstalk, and native cell–cell interactions, all of which can influence tumor radiosensitivity [71]. Patient‐derived organoids (PDOs) provide a more physiologically relevant preclinical platform because they retain tumor architecture, cellular heterogeneity, and patient‐specific molecular features and can be co‐cultured with stromal and immune cells to reconstruct aspects of the tumor microenvironment [72]. Comparative analyses of radiosensitive and radioresistant PDOs could further test the mechanisms proposed here, identify clinically relevant biomarkers, and facilitate development of personalized strategies to overcome radioresistance. In addition, single‐cell and spatial transcriptomic approaches may help resolve spatial heterogeneity within tumors and their surrounding microenvironments, an important contributor to clinical radioresistance.

The molecular links between intrinsic cellular features and cell‐fate decision post‐irradiation also remain incompletely defined. In particular, crosstalk among transcriptional regulation, chromatin architecture, and cell cycle progression requires further investigation. Because 3D genome structure is dynamically remodeled across the cell cycle, multilayer validation using clinical specimens will be important for translating these findings. Single‐cell Hi‐C (scHi‐C) is well suited to investigate how spatial genome organization influences radiosensitivity at single‐cell resolution [73]. High‐sensitivity methods for mapping DSBs, including DSBCapture, breaks labeling in situ and sequencing (BLISS), END‐seq, and INDUCE‐seq, may further clarify the interplay between 3D chromatin structure and radiation‐induced DNA damage [74, 75, 76, 77, 78]. However, single‐cell DSB‐mapping technologies remain under development and represent technical barriers for future work. Continued advances in multi‐omic methods should deepen our understanding of cancer radioresponse heterogeneity and support the development of improved radiotherapy strategies.

## 4 Conclusions

In conclusion, our study provides new insights into radioresponse heterogeneity among isogenic LUAD cells by integrating transcriptional and regulatory landscape. Building on these findings, we propose a radiosensitization strategy and identify several candidate molecular targets, laying the groundwork for mechanistic and translational studies to advance precision radiotherapy for lung adenocarcinoma.

## 5 Materials and Methods

### 5.1 Cell Culture and Radiation Treatment

The A549 cells (obtained from GeneChem), harboring wild‐type *TP53*, were cultured in Dulbecco's modified Eagle medium (DMEM; Gibco, C11005500BT) supplemented with 10% fetal bovine serum (FBS; PAN, ST30‐330) and 100 units/mL penicillin‐streptomycin solution (MAOKANG, MS0024) at 37°C in 5% CO_2_. Cell line identity was confirmed by short tandem repeat (STR) profiling (Supporting information). A single‐cell‐derived LUAD population was established by monoclonal culture. Cells were then irradiated with 6 Gy ^6^ ^0^Co γ‐ray at a dose rate of 61.42 cGy/min and used for subsequent single‐cell sequencing and experimental analyses.

### 5.2 Cell Proliferation

Cell proliferation was assessed using the Cell Counting Kit‐8 (CCK‐8) assay (Dojindo, CK04). Single‐cell‐derived LUAD cells at subconfluence were trypsinized (Gibco, 25200056), washed three times with phosphate‐buffered saline (PBS; Gibco, C10010500BT), and resuspended in DMEM supplemented with 10% FBS. Cells were seeded in 96‐well plates at 2000 cells/well in 200 µL medium. After 24 h, experimental cells received 6 Gy γ‐irradiation. At 0, 2, 6, 12, and 24 h post‐irradiation, the culture medium was replaced with 100 µL DMEM containing 10% CCK‐8 reagent, followed by incubation for 1.5 h at 37°C. Absorbance, reported as optical density at 450 nm (OD450), was measured using a Labsystems Multiskan microplate reader. Proliferation data are presented as the mean ± standard deviation (SD) from five independent experiments (*n* = 5). Multiple‐group comparisons were performed by one‐way analysis of variance (ANOVA) followed by Tukey's post hoc test in GraphPad Prism 9.5.0 (Windows). *p* < 0.05 was considered statistically significant. Significance markers were defined as follows: not significant (N.S.), *p* > 0.05; *p < 0.05; **p < 0.01; ***p < 0.001.

### 5.3 Immunofluorescence

Single‐cell‐derived LUAD cells (1 × 10^5^) were seeded on cell‐culture coverslips in 24‐well plates and cultured for 24 h before irradiation. At 2 h and 6 h post‐irradiation, experimental and control cells were washed with PBS and fixed in 4% paraformaldehyde for 15 min at room temperature (RT). After washing with PBS, cells were permeabilized with 0.3% Triton X‐100 plus 5% bovine serum albumin (BSA) in PBS (Solarbio, T8200; SW3015) for 20 min at RT, blocked with 1% BSA and 0.3% Triton X‐100 in PBS for 45 min at RT, and incubated overnight at 4°C with a 1:400 dilution of the γH2AX primary antibody (Cell Signaling Technology, 9718T/9718S). Cells were then washed and incubated for 45 min at RT with a 1:1000 dilution of goat anti‐rabbit secondary antibody (Thermo Fisher Scientific, F‐2765). Nuclei were counterstained using fluoroshield containing 4′,6‐diamidino‐2‐phenylindole (DAPI; Solarbio, S2110). Coverslips were mounted for confocal microscopy (SUNNY CSIM 100/110, 512 × 512 matrix). ImageJ was used to quantify fluorescence intensity and cell number [79], and normalized per‐cell fluorescence intensity was calibrated to the mean of the negative‐control group. Group differences were analyzed by one‐way ANOVA followed by Tukey's honestly significant difference (HSD) post hoc test in GraphPad Prism 9.5.0 (Windows).

### 5.4 Apoptosis Assay by Flow Cytometry

Single‐cell‐derived LUAD cells were seeded in six‐well plates at 3 × 10^5^ cells/well. After 24 h, cells in the experimental groups were exposed to 6 Gy γ‐ray, whereas control cells received no irradiation. At 2 h and 6 h after exposure, cells from both groups were harvested, double‐stained with 7‐aminoactinomycin D (7‐AAD) and allophycocyanin (APC)‐Annexin V (Biogems, 62700–80), and analyzed using a BD FACSCalibur flow cytometer. Apoptotic cells were defined as cells positive only for APC‐Annexin V staining. Statistical significance was assessed using a two‐tailed Student's t‐test. *p* < 0.05 was considered statistically significant. Significance markers were defined as follows: not significant (N.S.), *p* > 0.05; *p < 0.05; **p < 0.01; ***p < 0.001.

### 5.5 Cell Cycle by Flow Cytometry

Single‐cell‐derived LUAD cells (3 × 10^5^) were seeded in six‐well plates and incubated for 24 h. Cells in the experimental groups were then exposed to 6 Gy γ‐ray, whereas control cells received no irradiation. At 2 h and 6 h after exposure, irradiated and non‐irradiated cells were collected, washed with PBS, and fixed overnight at −20°C in 70% ice‐cold ethanol. Fixed cells were stained with propidium iodide (25 µg/mL) and ribonuclease A (RNase A; 2 µg/mL) from KeyGEN (KGA512) and analyzed using a BD FACSCalibur flow cytometer. The proportions of cells in G0/G1, S, and G2/M phases were determined by fitting the cell cycle distributions using ModFit software.

### 5.6 Single‐Cell RNA Sequencing and Single‐Cell ATAC Sequencing

For scRNA‐seq, three groups of LUAD cells were collected: single‐cell‐derived LUAD cells as the negative control (NC), and irradiated LUAD cells cultured for 2 h or 6 h post‐irradiation. For scATAC‐seq, three groups were collected: single‐cell‐derived LUAD cells (NC), cells harvested immediately after irradiation, and cells cultured for 2 h post‐irradiation. Samples for single‐cell analysis were prepared according to the 10x Genomics protocol. Briefly, 1 mL of each cell suspension was centrifuged in 1.5 mL tube at 400 × *g* for 5 min to obtain a cell pellet. The supernatant was removed, and the pellet was resuspended in PBS containing 0.04% BSA. Cells were washed twice by centrifugation at 150 × *g* for 3 min and then resuspended in approximately 500 µL PBS containing 0.04% BSA by gentle pipetting 10–15 times. Cell numbers were determined using an automated cell counter (LUNA II), and cell viability was assessed by 0.4% Trypan Blue staining. JingNeng Company performed library construction and sequencing using the 10x Genomics Chromium Controller and the single‐cell 3′ v2 kit for library preparation. scRNA‐seq and scATAC‐seq libraries were sequenced on NovaSeq 6000 PE153 and HiSeq 4000 PE74 platforms, respectively.

### 5.7 *KDM4B* Inhibition

The *KDM4B* inhibitor NCGC00244536 (HY‐101799; MedChemExpress, MCE) was used in this study. A 10 mM stock solution of NCGC00244536 was prepared in dimethyl sulfoxide (DMSO; Sigma‐Aldrich, D2438). For the experimental group, the stock solution was added to DMEM to obtain a final drug concentration of 10 µM. An equivalent volume of DMSO was added to the medium of the negative‐control group. All cells were incubated for 48 h before irradiation. At 6 h post‐irradiation, cell‐proliferation assays and immunofluorescence staining were performed as described above. The OD value at 6 h post‐irradiation was normalized to the corresponding OD value at 0 h for each group.

Detailed bioinformatic pipelines, including analyses of scRNA‐seq and scATAC‐seq data, and assessment of the effects of *TP53* knockout and mutation, are described in the Supporting Information Methods.

## Author Contributions

Huan Tao was responsible for data analysis, experimental implementation, manuscript drafting, and revision. Yaru Li took part in data analysis and manuscript revision. Leixin Ge performed additional supporting experiments. Yu Sun and Longteng Wang assisted with data analysis. Lu Chen and Feiyue Ma conducted laboratory experiments. Yang Zheng was in charge of figure preparation and revision. Junting Wang and Xuemei Bai revised the manuscript. Changhui Ge, Hanjiang Fu, Hao Li, Hebing Chen, Xiaochen Bo, and Xiaofei Zheng supervised this study and provided critical guidance for the research design. Hebing Chen and Xiaofei Zheng obtained the financial support for this work. All authors have read and approved the final manuscript.

## Funding

This work was supported by the National Key Research and Development Program of China (No. 2023YFF0725500 to H.C.), the National Natural Science Foundation of China (http://www.nsfc.gov.cn; Nos. 32071290 and 91540202 to X.Z.; No. 62422318 to H.C.), Beijing Nova Program of Science and Technology (20250484974 to H.C.), and the State Key Laboratory of Medical Proteomics (SKLP‐K202407 to H.C.).

## Ethics Statement

The authors have nothing to report.

## Conflicts of Interest

The authors declare no conflicts of interest.

## Supporting information




**Supporting File 1**: mco271071‐sup‐0001‐SuppMat.docx.

**Supporting File 2**: mco271071‐sup‐0002‐Tables.xlsx.

## Data Availability

The scRNA‐seq data and scATAC‐seq data of this study have been deposited in the Gene Expression Omnibus under accession code GSE211617. Processed mRNA‐expression and clinical data for LUAD patients were downloaded from The Cancer Genome Atlas (TCGA) database in April 2022. All TCGA data utilized in this work correspond to open‐access tiers. The data for 107 radiotherapy‐treated LUAD patients were filtered from the downloaded local dataset. Processed data can be explored through figshare (https://doi.org/10.6084/m9.figshare.27153453). The code used for the analysis is publicly available at GitHub (https://github.com/liyarubio/Dissecting‐the‐radioresponse‐heterogeneity).
