# Supplementary material for: Dissecting the Radioresponse Heterogeneity of Lung Adenocarcinoma Cells From the Perspective of Transcriptional and Regulatory Landscape
Source: MedComm (2020). 2026 Oct 21;7(11):e71071. doi: 10.1002/mco2.71071 (PMC13686437; doi:10.1002/mco2.71071)
Supplement: Supplementary file 1 [file MCO2-7-e71071-s001.docx]

# Dissecting the radioresponse heterogeneity of lung adenocarcinoma cells from the perspective of transcriptional and regulatory landscape

**Running title:** The radioresponse heterogeneity of LUAD cells

Huan Tao ^1, #^, Yaru Li ^1, #^, Leixin Ge ^1, #^, Lu Chen ^1^, Yang Zheng ^1^, Feiyue Ma ^1^, Changhui Ge ^1^, Hanjiang Fu ^1^, Yu Sun ^1^, Longteng Wang ^2^, Junting Wang ^1^, Xuemei Bai ^1^, Hao Li ^1^, Xiaochen Bo ^1,^ *, Hebing Chen ^1,^ * and Xiaofei Zheng ^1,^ *

1. Academy of Military Medical Sciences, Beijing 100850, China
2. Center for Statistical Science, Center for Bioinformatics, School of Life Sciences, Peking University, Beijing 100871, China

^#^ These authors contributed equally to this work.

* Correspondcance: [xfzheng100@126.com](mailto:xfzheng100@126.com) (Xiaofei Zheng), chenhb@bmi.ac.cn (Hebing Chen) and boxc@bmi.ac.cn (Xiaochen Bo).

### Supplemental materials

### Supplementary tables

All complete numerical values or content for Supplementary Tables S1-S7 are provided in the separate Excel file.

Table S1 Quality control metrics of single-cell RNA sequencing (scRNA-seq) data

Table S2 Quality control metrics of single-cell assay for transposase-accessible chromatin with high-throughput sequencing (scATAC-seq) data. IR: ionizing radiation.

Table S3 Peak markers in different regulatory cell states of lung adenocarcinoma (LUAD) cells

Table S4 Gene markers highly expressed in radiation-associated transcriptional cell state

Table S5 Clinicopathological characteristics of 107 LUAD patients with radiation therapy from The Cancer Genome Atlas (TCGA) database

Table S6 Specific information of LUAD patients

Table S7 Scaled regulon activity in LUAD cells at 2 h post-irradiation

### Supplementary figures

FIGURE S1 | Quality control of single-cell RNA sequencing (scRNA-seq) data. Quality control metrics of scRNA-seq data performed by scCANCER, including droplets number, unique molecular identifiers (UMIs), the number of expressed genes, and the percentage of transcripts from mitochondrial, ribosomal and dissociation-associated genes.

FIGURE S2 | Protein-protein interaction network in cluster 10. (A) Connected network components in protein-protein interaction network identified in marker genes of cluster 10. (B) The three best-scoring functional descriptions (P value) of the corresponding components. The P values were calculated based on the cumulative hypergeometric distribution and terms with P value < 0.01 were collected. Log10 (P) is the P value in log base 10.

FIGURE S3 | Quality control of single-cell assay for transposase-accessible chromatin with high-throughput sequencing (scATAC-seq) data. (A) Histograms of fragments' number per barcode for non-cells and cells. (B) Insert size distribution in linear scale. (C) Enrichment score of transcription start site (TSS). The TSS profile was the summed accessibility signal (defined as number of cut sites per base) in a window of 2,000 bases around all the annotated TSSs, normalized by the minimum signal in the window. This metric reported the maximum value in the profile. (D) Targeting scatter plot. Each dot represented a barcode. Horizontal axis was the barcode's number of fragments, vertical axis was the percentage of those fragments that overlap peaks. Non-cell and cell groups were represented with different colors.

FIGURE S4 | Radiation-associated transcriptional cell state. (A) Quantification of immunofluorescence for phosphorylated histone H2AX (γH2AX). The values were expressed as the mean ± standard deviation (SD) of at least three replicates and the data was tested for statistically significant differences between groups using one-way analysis of variance (ANOVA), followed by Tukey HSD post hoc test. Significance markers were defined as follows: not significant (N.S.), P > 0.05; *, P < 0.05; **, P < 0.01; ***, P < 0.001. (B) Number of lung adenocarcinoma (LUAD) cells in each cluster at 2 h and 6 h post-irradiation, color-coded by sample. (C) Ionizing radiation (IR)-associated relative likelihood of irradiated cells in each cluster at 6 h post-irradiation calculated by MELD, which were expressed as the mean ± SD. (D) Proportion of cells in different cell cycle in cluster 12, evaluated by SCRAN, colour-coded by cell cycles. (E) Volcano plot showed the differentially expressed genes between cluster 5 and cluster 12 LUAD cells at 6 h post-irradiation (P < 0.05, red-colored up-regulated genes and blue colored down-regulated genes). P-values were obtained from the Wilcoxon Rank Sum test with Bonferroni correction. (F) Gene Ontology (GO) analysis of highly expressed genes in cluster 5 compared to cluster 12 at 6 h post-irradiation.

FIGURE S5 | Pseudo-bulk analysis of scATAC-seq data. (A) Volcano plot showed the differentially accessible transcription factors (TFs) in LUAD cells at different study time post-irradiation, based on scATAC-seq data (P < 0.05, red colored up-regulated TFs and blue colored down-regulated TFs). P values were obtained from the moderated t-statistic, after adjustment for multiple testing (Benjamini and Hochberg's method). (B) Motifs of TFs less accessible post-irradiation.

FIGURE S6 | Regulatory topics identified by cisTopic based on scATAC-seq data. (A) Optimal number of topics was selected based on the highest log-likelihood in the last iteration. (B-C) Heatmap (B) and t-SNE (C) view show the region annotation of 12 topics, including the region type and the closest gene. t-SNE view was visualized based on region-topic distributions. (D) Top enriched motifs in topic 1 with normalized enrichment score. t-SNE view was visualized based on cell-topic distributions. POU class 4 homeobox 1 (POU4F1), DEAF1 transcription factor (DEAF1), GATA binding protein 1 (GATA1), Yin Yang 1 transcription factor (YY1) E2F transcription factor 1 (E2F1), NK2 homeobox 8 (NKX2-8).

### Supplementary methods

### Upstream analysis of scRNA-Seq and scATAC-seq data

To analyze scRNA-seq data, the processing steps included sample demultiplexing, barcode handling, and quantification of unique molecular identifiers (UMIs) using the CellRanger of 10x Genomics. Default parameters were utilized for this analysis. The initial step involved demultiplexing raw reads from each library using both 'cellranger mkfastq' and 'bcl2fastq'. This process generated two fastq files: one consisted of 26-bp reads with cell barcodes and UMIs, while the other contained 96-bp reads with cDNA sequences. Subsequently, alignment to the human genome reference (GRCh38), filtering, and counting were performed using 'cellranger count' to establish the gene-barcode matrix. Detailed statistics regarding barcoding and sequencing outcomes could be found in Table S1. For scATAC-seq data analysis, single-cell accessibility counts were determined with 'cellranger-atac count'. Summary information on scATAC-seq data metrics was presented in Table S2.

### Quality control

Expression matrices from scRNA-seq data underwent processing with scCANCER (v2.2.1) [1]. The elimination of droplets capturing lysed cells, doublets, or with low transcript diversity was conducted using five metrics: total UMIs, expressed gene count, and mitochondrial, ribosomal, and dissociation-associated gene UMI percentages per cell [1]. The scCANCER doublet score was computed using the scds (v1.2.0) algorithm, which combines binary classification and co-expression data [1]. Following quality control, downstream analyses were carried out with Seurat (v4.1.0) by applying canonical correlation analysis (CCA) to integrate scRNA-seq data and address batch effects across samples [1, 2]. For scATAC-seq data, per-cell quality control assessments were performed utilizing DNA accessibility metrics, such as nucleosome banding pattern strength and transcriptional start site (TSS) enrichment score. Signac (v1.5.0) was used to identify and exclude low-quality cells based on these measurements [3].

### Dimensionality reduction and integration of scRNA-seq data

Expression matrices from different scRNA-seq data were integrated and log-normalized using Seurat [2]. 'FindVariableGenes' was applied to identify highly variable genes [2]. Then, the highly variable genes were used as input to produce the t-Distributed Stochastic Neighbour Embedding (t-SNE) projection and Uniform Manifold Approximation and Projection (UMAP) using the 'RuntSNE' (perplexity=30, theta=0.5, maximum iteration=1000, learning rate=250, and momentum reduction=0.5) and 'RunUMAP' (min_dist=0.3, metric=correlation, n_neighbors=30) [2].

### Quantifying the radiation response of LUAD cells by MELD

We performed MELD (v1.0) to quantify the radiation response at the single-cell level using scRNA-seq data [4]. Relative likelihood (RL), the effect of radiation on each cell, was calculated by comparing the transcriptomic space between non-irradiated and irradiated cells. Based on the relative likelihood, irradiated cells were manually classified into high-, moderate-, and low- responsive cells to γ-ray treatment. Cells with RL greater than or equal to 0.7 were classified as high-responsive cells, cells with RL greater than 0.5 but less than 0.7 were classified as moderate-responsive cells, and cells with RL less than or equal to 0.5 were classified as low-responsive cells.

### TCGA data

The mRNA expression profiling data of 513 LUAD samples was downloaded from The Cancer Genome Atlas database in April 2022 (TCGA, <https://tcga-data.nci.nih.gov/tcga/),> all TCGA data used in this study are open access. After excluding patients without radiotherapy and complete survival information, 107 patients were used for the following analysis.

### Radiosensitivity index

Radiosensitivity index was calculated by 10-hub-gene assay on 107 LUAD samples. The RSI value was then determined using the previously published linear regression algorithm:

RSI = -0.0098009 × AR + 0.0128283 × JUN + 0.0254552 × STAT1 - 0.0017589 × PRKCB - 0.0038171 × RELA + 0.1070213 × ABL1 - 0.0002509 × SUMO1 - 0.0092431 × CDK1 - 0.0204469 × HDAC1 - 0.0441683 × IRF1 [5]. Patients were divided into radioresistant and radiosensitive groups using the 50th percentile RSI as the cutoff. The dichotomization was set at 0.225, with RSI ≥ 0.225 representing radioresistant and RSI < 0.225 representing radiosensitive. The statistical significance was determined by two-tailed Student's t-test. All results with p < 0.05 were considered statistically significant. Significance markers were defined as follows: not significant (N.S.), P > 0.05; *, P < 0.05; **, P < 0.01; ***, P < 0.001.

### The effect of *TP53* knockout and mutation on the expression of key genes in radiation-associated transcriptional cell state

RNA-seq raw sequencing data were downloaded from the Gene Expression Omnibus (GEO) repository under accession GSE262053. This dataset comprises a panel of isogenic A549 cell lines with distinct *TP53* genotypes: wild-type (WT), *TP53*-knockout (KO), and two hotspot mutant variants (R175H and R273H). Only DMSO-treated samples were retained for subsequent analysis to characterize baseline transcriptional landscapes across all four *TP53* backgrounds. Gene-level raw read counts were normalized with the Bioconductor package edgeR [6], then transformed to log₂(CPM+1) for downstream expression plotting. Prior to heatmap generation, average gene expression values were computed from biological replicates grouped by each *TP53* genotype. All biological replicates were treated as independent observations throughout statistical analysis. The statistical significance was determined by two-tailed student t-test. Significance markers were defined as follows: not significant (N.S.), P > 0.05; *, P < 0.05; **, P < 0.01; ***, P < 0.001.

### Cell cycle assignment

Based on gene expression data, we assigned cells to their cell cycle phases using SCRAN (v1.18.7), which can predict cell cycle from scRNA-seq based on paired markers changing across cell cycle phases [7]. Specifically, cell cycle was assigned by calculating phase score of each cell. Cells are assigned to the G1 phase if their G1 score is greater than 0.5 [7]. Similar to G1, if G2 score exceeds 0.5, cells are assigned to the G2/M phase. If both G1 and G2/M scores are above 0.5, the cell is assigned to the phase corresponding to the higher score [7]. Cells were assigned to S phase with G1 and G2/M scores below 0.5 [7]. The P values are calculated using two independent sample Z-test. All results with P < 0.05 were considered statistically significant. Significance markers were defined as follows: not significant (N.S.), P > 0.05; *, P < 0.05; **, P < 0.01; ***, P < 0.001.

### Pseudo-bulk analysis

To perform pseudo-bulk analysis of scATAC-seq data, we integrated three scATAC-seq datasets using Signac (v1.5.0) and Seurat, and computed differentially accessible TFs using chromVAR (v1.12.0) [3, 8].

### Trajectory inference analysis

Partition-based graph abstraction (PAGA) offers an interpretable graph-like map of the arising data manifold, by assessing the connectivity of manifold partitions [9]. We analyzed the differential trajectory of different cell cycles of irradiated and non-irradiated LUAD cells using PAGA-tree and executed by Dyno, a uniform interface to trajectory inference methods [10]. We plotted key milestones for cell cycle transition in LUAD cells at 6 h post-irradiation and analyzed key genes important for transcriptomic transition between different milestones.

### Cell communication analysis

Intercellular communication networks between different cell cycle were inferred from scRNA-seq data using CellChat (v1.1.1) [11]. The resultant cell cycle labels inferred by SCRAN were applied [7]. Based on the manually curated signaling ligand-receptor interactions database, CellChat inferred cell-cycle specific signaling communications within irradiated and non-irradiated scRNA-seq data of LUAD cells based on the Seurat-normalized expression matrices using mass action models, along with differential expression analysis and statistical tests on different cell cycle groups.

### RNA velocity analysis

The conversion of bam files to loom files containing spliced, unspliced, and undefined matrices was performed using Velocyto.py (v0.17.17) [7, 12]. Subsequently, the loom files were transformed into a Seurat object in R, integrating the cell cycle labels analyzed by SCRAN. The calculation and visualization of RNA velocity were achieved using dynamo (v 1.0.0) [7].

### Identification of DEGs and gene enrichment analysis

Differentially expressed genes (DEGs) between two clusters were identified by FindMarkers function in Seurat [2]. The test was performed using the Wilcoxon Rank Sum test with Bonferroni correction. The genes with adjusted P < 0.05 are considered to be DEGs. Gene ontology analyses were performed using Metascape [13]. The P values for each ontology category are calculated based on the cumulative hypergeometric distribution.

### Identification of radiation-associated regulatory cell state based on scATAC-seq data

scATAC-seq data were firstly integrated by 'cellranger atac aggr'. Radiation-associated regulatory cell state was identified by cisTopic based on integrated scATAC-seq data [14]. 12 topics were selected as the best predicted result by 'selectModel'. Topics were annotated by “hg19-regions-1M-9species.all_regions.mc9nr.feather” in Rcistarget database. 'liftOver=hg38_to_hg19_list' was used to convert genome coordinates to fit annotation file. Two kinds of t-SNE were calculated using wrapper functions included in cisTopic based on cell-topic distribution and region-topic distribution [14]. In this case, 'cell' and 'region' are used as 'target' parameter in 'runtSNE', respectively. Only high confidence regions (i.e. that pass the binarization threshold at least in 1 topic) are used in the calculation of region-based t-SNE [14].

### Regulatory network analysis

The scRNA-seq data was analyzed using pySCENIC (v0.11.2), with the expression profiles fed into derive co-expression modules from the grn, ctx, and aucell matrices [15]. Enriched motifs for a gene signature were identified as a regulon. The activity of regulons was measured by AUCell function and the average regulon activity was calculated for high-, moderate- and low-responsive LUAD cells at 2 h post-irradiation. Subsequently, GRNBoost was employed to reconstruct the gene regulatory network which gave an importance representing how specific TF regulated candidate regulatory genes [16, 17]. Then the importance was normalized by ‘importArboreto()’ command and the top 50 regulatory genes of POLR2A were visualized using Cytoscape v3.8.2 [16-18].

### Reference

1. Guo, W., Wang, D., Wang, S., et al., "scCancer: a package for automated processing of single-cell RNA-seq data in cancer," Brief Bioinform 22, no. 3 (2021): bbaa127.

2. Hao, Y., Hao, S., Andersen-Nissen, E., et al., "Integrated analysis of multimodal single-cell data," Cell 184, no. 13 (2021): 3573-3587.e29.

3. Stuart, T., Srivastava, A., Madad, S., et al., "Single-cell chromatin state analysis with Signac," Nat Methods 18, no. 11 (2021): 1333-1341.

4. Burkhardt, D.B., Stanley, J.S., 3rd, Tong, A., et al., "Quantifying the effect of experimental perturbations at single-cell resolution," Nat Biotechnol 39, no. 5 (2021): 619-629.

5. Dai, Y.H., Wang, Y.F., Shen, P.C., et al., "Radiosensitivity index emerges as a potential biomarker for combined radiotherapy and immunotherapy," NPJ Genom Med 6, no. 1 (2021): 40.

6. McCarthy, D.J., Chen, Y. and Smyth, G.K., "Differential expression analysis of multifactor RNA-Seq experiments with respect to biological variation," Nucleic Acids Res 40, no. 10 (2012): 4288-97.

7. Qiu, X., Zhang, Y., Martin-Rufino, J.D., et al., "Mapping transcriptomic vector fields of single cells," Cell 185, no. 4 (2022): 690-711.e45.

8. Schep, A.N., Wu, B., Buenrostro, J.D., et al., "chromVAR: inferring transcription-factor-associated accessibility from single-cell epigenomic data," Nat Methods 14, no. 10 (2017): 975-978.

9. Wolf, F.A., Hamey, F.K., Plass, M., et al., "PAGA: graph abstraction reconciles clustering with trajectory inference through a topology preserving map of single cells," Genome Biol 20, no. 1 (2019): 59.

10. Saelens, W., Cannoodt, R., Todorov, H., et al., "A comparison of single-cell trajectory inference methods," Nat Biotechnol 37, no. 5 (2019): 547-554.

11. Jin, S., Guerrero-Juarez, C.F., Zhang, L., et al., "Inference and analysis of cell-cell communication using CellChat," Nat Commun 12, no. 1 (2021): 1088.

12. La Manno, G., Soldatov, R., Zeisel, A., et al., "RNA velocity of single cells," Nature 560, no. 7719 (2018): 494-498.

13. Zhou, Y., Zhou, B., Pache, L., et al., "Metascape provides a biologist-oriented resource for the analysis of systems-level datasets," Nat Commun 10, no. 1 (2019): 1523.

14. Bravo González-Blas, C., Minnoye, L., Papasokrati, D., et al., "cisTopic: cis-regulatory topic modeling on single-cell ATAC-seq data," Nat Methods 16, no. 5 (2019): 397-400.

15. Van de Sande, B., Flerin, C., Davie, K., et al., "A scalable SCENIC workflow for single-cell gene regulatory network analysis," Nat Protoc 15, no. 7 (2020): 2247-2276.

16. Shannon, P., Markiel, A., Ozier, O., et al., "Cytoscape: a software environment for integrated models of biomolecular interaction networks," Genome Res 13, no. 11 (2003): 2498-504.

17. Aibar, S., González-Blas, C.B., Moerman, T., et al., "SCENIC: single-cell regulatory network inference and clustering," Nat Methods 14, no. 11 (2017): 1083-1086.

18. Moerman, T., Aibar Santos, S., Bravo González-Blas, C., et al., "GRNBoost2 and Arboreto: efficient and scalable inference of gene regulatory networks," Bioinformatics 35, no. 12 (2019): 2159-2161.

STR profile report
